# Transcriptome Analysis of Rainbow Trout (*Oncorhynchus mykiss*) Eggs Subjected to the High Hydrostatic Pressure Treatment

**DOI:** 10.1155/2018/5197126

**Published:** 2018-08-26

**Authors:** Artur Gurgul, Klaudia Pawlina-Tyszko, Monika Bugno-Poniewierska, Tomasz Szmatoła, Igor Jasielczuk, Stefan Dobosz, Konrad Ocalewicz

**Affiliations:** ^1^Laboratory of Genomics, Department of Animal Genetics and Molecular Biology, National Research Institute of Animal Production, Krakowska 1, 32-083 Balice, Poland; ^2^Institute of Veterinary Sciences, University of Agriculture in Krakow, Mickiewicza 24/28 Av, 30-059 Krakow, Poland; ^3^Department of Salmonid Research, Inland Fisheries Institute in Olsztyn, Rutki, 83-330 Żukowo, Poland; ^4^Department of Marine Biology and Ecology, Institute of Oceanography, Faculty of Oceanography and Geography, University of Gdansk, M. Piłsudskiego 46 Av, 81-378 Gdynia, Poland

## Abstract

High hydrostatic pressure (HHP) causes depolymerization of the spindle microtubules. HHP applied to fish eggs prevents extrusion of the second polar body and inhibits the first cell cleavage, and it is used to produce triploids and diploid gynogenetic and androgenetic individuals. HHP has been also found to affect biomolecules including nucleic acids, and it may be presumed that HHP administered to the rainbow trout (*Oncorhynchus mykiss*) eggs disturbs cytoplasmic maternal RNA indispensable for the early embryogenesis. To verify this assumption, quality and quantity of RNA extracted from the rainbow trout eggs subjected to the high hydrostatic pressure shock were analyzed. Provided results exhibited that maternal transcriptome was resistant to a three-minute exposure to 65.5 MPa of HHP treatment. Some trend showing increase of the RNA integrity was observed in the HHP-treated eggs; however, the difference was not statistically significant. Alterations in the expression profiles in the rainbow trout eggs subjected to HHP were also negligible. Greater differences in the maternal gene expression were observed between eggs from different clutches than between HHP-treated and untreated eggs from the same clutch. It may be assumed that exposure to HHP shock was too short to modify significantly maternal transcripts in the rainbow trout eggs.

## 1. Introduction

High hydrostatic pressure (HHP) treatment may be lethal for the prokaryotic and eukaryotic cells. HHP about 200 MPa causes apoptotic death of the mammalian cells while treatment with HHP higher than 300 MPa results in the cell death by necrosis [1]. HHP inactivates many pathogens and keeps unchanged functional and nutritional characteristics of many products. Hence, it has been widely used in the food industry to inactivate microorganisms [2, 3]. Moreover, HHP might be applied for sterilization and disinfection of biomaterials, modulation of enzymatic activities, or genetic transformations, among others [1]. It has been observed that mammalian cells subjected to HHP of 40–60 MPa are more resistant to the cryopreservation process and thawed oocytes, spermatozoa, and embryos show increased stress tolerance and the postthaw survival [4, 5].

HHP causes depolymerization of the spindle microtubules in the fish eggs what impairs chromosome movement and disturbs meiotic and mitotic divisions. HHP shock implemented to fish eggs shortly after insemination with normal or UV-irradiated spermatozoa results in abortion of the 2nd polar body extrusion and enables development of the triploid or diploid meiogynogenetic individuals, respectively [6]. In the gamma-irradiated and inseminated eggs or eggs activated by the UV-irradiated spermatozoa, HHP applied at the time of prophase of the 1st zygotic division inhibits the cell cleavage which leads to duplication of the haploid set of chromosomes and production of the androgenetic and mitogynogenetic doubled haploids (DHs), respectively [7]. Apart from the cellular organelles [8, 9], HHP has been also found to affect DNA-protein complexes [10] and transcriptomes [11–13]. In fish, maternal mRNA deposited in the oocyte cytoplasm during oogenesis controls early embryonic development before activation of the zygotic genome which takes part at cell cycle 10 [14]. Thus, hydrostatic pressure shock administered to the rainbow trout eggs has been assumed to affect cytoplasmic maternal RNA indispensable for the early embryogenesis. To examine such assumption, quality and quantity of RNA isolated from rainbow trout (*Oncorhynchus mykiss*) eggs subjected to a three-minute exposure to 65.5 MPa of HHP were analyzed. Obtained results exhibited that rainbow trout maternal transcriptome was resistant to the HHP shock. Analysis of the transcriptome integrity did not reveal any statistically significant differences between HHP-treated and nontreated eggs. Alterations in the expression profiles of genes related to the fish development and growth, response to the DNA damage, actin filament polymerization, and function of the spindle microtubules observed in the HHP-treated eggs were not substantial. Interestingly, differences in the maternal gene expression were greater between eggs from different clutches than between HHP-treated and untreated eggs originated from an individual female.

## 2. Material and Methods

### 2.1. Gamete Collection and Egg Treatment

An experiment performed in 18 November 2015 was approved by the Animal Experiments Local Committee in Gdansk, Poland (no. 28/2015). Eggs originated from the winter spawning rainbow trout (*Oncorhynchus mykiss* Walbaum 1792) from the broodstock raised in the Department of Salmonid Research, Inland Fisheries Institute in Olsztyn, Rutki, Poland. In the case of rainbow trout from the studied broodstock, a three-minute HHP shock of 65.5 MPa was applied to the eggs 35 min after activation (early shock) or 350 min after activation (late shock) efficiently inhibits release of the 2nd polar body or prevents the 1st cell cleavage, respectively [15]. Before handling, the fish were anesthetized with Propiscin (etomidate, IRŚ, Poland) at a dose of 0.5 ml·l^−1^ of water. Three rainbow trout females were stripped and eggs were collected into the separate plastic bowls. In the present research, portions of 100 eggs from each female were shocked with 65.5 MPa for 3 min using TRC-APV electric/hydraulic apparatus (TRC Hydraulics Inc., Dieppe, Canada). Both HHP-treated and untreated (control) eggs were stored for 15 min at +4°C and then divided into two batches and either frozen on the dry ice or placed in RNA stabilization solution for tissues (RNAlater®) (Sigma-Aldrich), incubated overnight at +4°C, and finally stored at −80°C for further use.

### 2.2. RNA Purification and Degradation Analysis

RNA was isolated from the untreated eggs and eggs exposed to HHP originated from three females in five technical replicates including different methods of egg conservation and homogenization. For RNA extraction, eggs were thawed on ice and homogenized in TRIzol reagent (Thermo Fisher Scientific) using a manual and two homogenizer methods, Bullet Blender (bead mill) homogenizer (Next Advance) and TissueRuptor (Qiagen), in order to find the most efficient approach to provide high-quality RNA and to estimate the level of method-specific homogenization-induced RNA degradation. Two eggs were used for each replicate. Extracted RNA was then purified using a modified TRIzol procedure established at Igor Babiak Laboratory (University of Nordland, Bodø, Norway). The purified RNA was quantified using the NanoDrop2000 spectrophotometer (Thermo Fisher Scientific) and assessed for the quality using the Agilent 2200 TapeStation system (RNA screen tapes). The Agilent RIN (RNA integrity number) algorithm was used for the comparisons of RNA integrity between HHP-treated and untreated eggs. The algorithm analyzed not only the 28S and 18S rRNA ratios but also a whole electrophoretic trace of RNA samples, which includes the presence and absence of the degradation products [16]. RIN was standardized to be used for the comparative studies [17].

### 2.3. A Whole Transcriptome Sequencing and Data Analysis

Before sequencing library construction, a total RNA was additionally purified using Agencourt RNAClean XP beads (Beckman Coulter) according to the manufacturer protocol. A total of 800 ng purified RNA was used as an input for TruSeq RNA Sample Prep v2 kit (Illumina). Standard library construction steps (mRNA selection, fragmentation, cDNA synthesis, end repair, adenylation, indexed adapter ligation, and amplification) were followed by a qualitative (Agilent TapeStation 2200) and quantitative (Qubit, Thermo Fisher Scientific) evaluation. Validated and normalized libraries were eventually sequenced in a single 50 bp run (1 × 50 bp) on the HiScanSQ system using TruSeq SBSv3 Sequencing kit (Illumina) to obtain approx. 25 million reads per sample.

The obtained demultiplexed raw reads were filtered using the Flexbar software [18] to trim accidental sequences of adapters and remove the low-quality reads. The resulting read set was mapped against the newest available and supplemented rainbow trout transcriptome (encompassing 44,990 transcripts; downloaded from http://www.animalgenome.org/repository/pub/MTSU2014.1218/) [19] using Bowtie aligner [20], permitting for unlimited multimappings (−a). The obtained sample files were analyzed using eXpress (http://bio.math.berkeley.edu/eXpress/overview.html) software which can be used to estimate transcript abundances in the multi-isoform genes, and it is also able to resolve multimappings of reads across the gene families and does not require a reference genome so that it can be used even in the conjunction with de novo transcriptome assemblies. The underlying model is based on the previously described probabilistic models developed for RNA-seq [21] but it is also applicable to other settings where target sequences are sampled and includes parameters for the fragment length distributions, errors in reads, and sequence-specific fragment bias [22]. The obtained estimated rounded effective counts for separate transcripts and samples were put into a DESeq2 [23] software, which is a count-based differential expression analysis tool.

## 3. Results

The assigned RIN was independent of the sample concentration. No clear evidence for the RNA degradation was detected in the rainbow trout eggs exposed to 65.5 MPa of HHP shock that lasted 3 minutes. The mean observed RIN for all 15 replicates in control egg RNA was 8.44 (±0.58) and was similar to that observed for the eggs subjected to HHP (8.69 ± 0.47) (Table 1, Supplementary Figure S1). The paired *t*-test for group means exhibited some trend (*p* = 0.072), unexpectedly showing a slightly higher RNA integrity in the HHP-treated eggs. No significant differences (*p* = 0.380) have been found in the quantity of provided RNA when mean concentrations of RNA extracted from the eggs exposed to HHP and untreated eggs were similar (191.2 ± 93.2 versus 173.3 ± 34.6 ng/*μ*l, resp.). The detailed statistics on RNA integrity and concentrations are presented in Supplementary Table S1.

**Figure 1:**
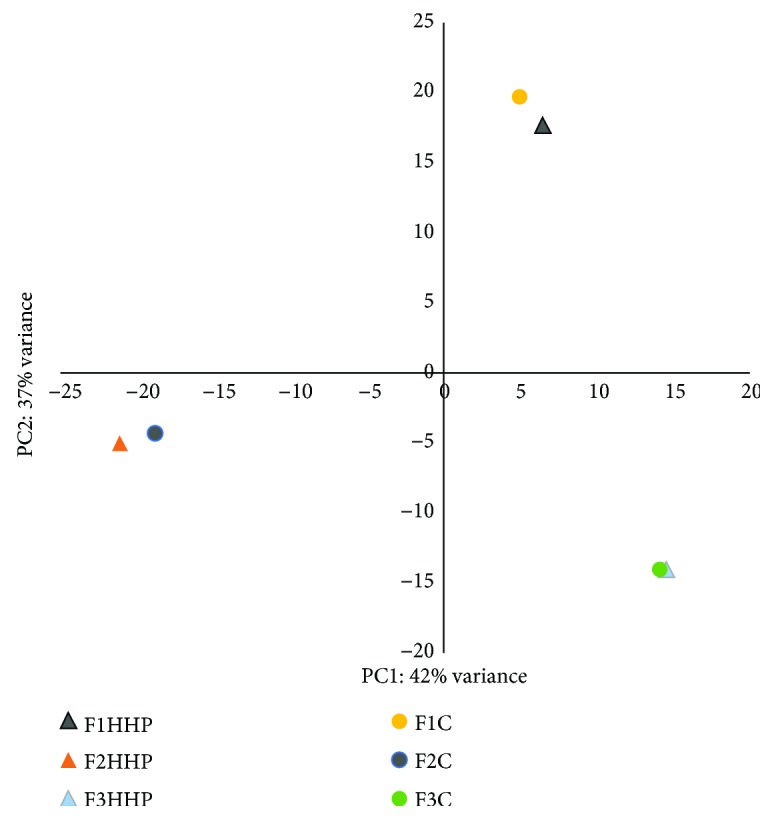
Principal component analysis (PCA). RNA-seq expression data from the rainbow trout eggs treated with high hydrostatic pressure (HHP) and untreated control eggs (C). Female (F1–F3) eggs that were treated with HHP are marked with triangles, and their untreated eggs (C) are marked with dots.

In total, 162.2 million sequencing reads were obtained and about 60% of them were successfully mapped to the reference transcriptome (Supplementary Table S1). eXpress software was used to estimate transcript abundance in each sample. In the control eggs 16,346 expressed transcripts were detected with at least one RPKM (reads per kilobase of exon per million reads mapped). A similar number of transcripts (16,243) were detected in the HHP-treated eggs. The obtained effective read counts analyzed with DESeq2 showed no significantly altered transcript levels (after FDR correction from *p* values using the Benjamini-Hochberg procedure) (Supplementary File Data). Examination of the expression patterns with the principal component analysis (PCA) showed expression profile clustering according to the individual from which eggs were taken but not according to experimental conditions (Figure 1). PCA also showed some interindividual variation with first two PCA components explaining 42 and 37% of variance, respectively. Pearson's correlation between expression profiles of untreated eggs in the pairwise comparisons for the separate individuals was high and ranged from 0.977 to 0.997.

The top 10 transcripts with the lowest pointwise *p* value (*p* < 0.01) for the difference in the expression level between HHP-treated and untreated eggs encompassed transport protein *Sec31A*-like *Oreochromis niloticus* homolog, insulin-like growth factor 1b receptor *Oncorhynchus kisutch* homolog, telomere-associated protein *RIF1 Oreochromis niloticus* homolog, *IGF-I* receptor subtype A, *FH1/FH2* domain-containing protein 3 (*Danio rerio* homolog), domain-containing glycophosphatidylinositol anchor protein, centromere protein F (*Oreochromic niloticus* homolog), *FH1/FH2* domain-containing protein 3 (*Oreochromis niloticus* homolog), histone-lysine N-methyltransferase *SETD2*-like (*Oreochromis niloticus* homolog), and male-specific lethal 3-like 1 (*Salmo salar* homolog) (Table 2, Supplementary File Data). However, RNA-seq results presented here should be confirmed using quantitative RT-PCR approach to verify differences between HHP-treated and control eggs.

## 4. Discussion

Induced development of triploids and diploid gynogenetic and androgenetic fish includes 1 to 10 min exposure of activated eggs to HHP of 48.3 to 79.3 MPa in order to damage spindle microtubules and to disrupt extrusion of the 2nd polar body or to inhibit 1st cell cleavage [6, 7]. However, short HHP treatment of fish eggs does not have to affect cytoplasmic maternal mRNA that is indispensable for the early embryonic development in fish. The three-minute exposure to 65.5 MPa of HHP that has been usually applied to recover diploid state in the rainbow trout eggs during gynogenesis [15] did not degrade maternal transcripts. It is not excluded that longer HHP treatment might impair stability of the maternal mRNA as it has been observed in the pig oocytes after one-hour exposure to 20 MPa of hydrostatic pressure [24]. However, such long HHP treatment might be damaging for the fish eggs.

High integrity of RNA in rainbow trout eggs subjected to HHP shock suggests such treatment has not decreased developmental competence of the eggs. HHP treatment prior cryopreservation increased stress tolerance and improved survival rates, fertilizing ability, and development competence of the vitrified porcine oocytes [4, 5]. It has been found that increased stress tolerance in the mammalian oocytes pretreated with the sublethal HHP results from the induction of the posttranscriptional activation of the shock proteins [4]. Bovine embryos treated with 40 and 60 Mpa of HHP exhibited increased embryo competence through downregulation of genes connected to cell death and apoptosis process and upregulation of genes responsible for the RNA processing, cellular growth, and proliferation [12]. In turn, higher dose of HHP (80 MPa) decreased embryo competence that showed downregulated cell cycle-related genes and upregulated genes engaged in the apoptosis mechanism [12].

Many studies performed so far using RNA-seq method are based on the three replicates per treatment that is accepted as minimal number of samples needed for analysis. Although three biological replicates is not many and the obtained statistical power may be low, the expression changes with high fold change and high uniformity of change across replicates should be reliably identified. In our study, profiles of expression transcripts in the HHP-treated rainbow trout eggs were altered when compared to those in the untreated eggs; however, the differences were not significant at the genome-wide level. This may be explained by the large expression differences between eggs from the different females. Indeed, PCA of expression profiles in the rainbow trout eggs exhibited stronger similarity between gametes from the individual female rather than from the experimental conditions (Figure 1). This observation suggests that interindividual differences between rainbow trout females from the winter broodstock had larger influence on the egg transcriptome and developmental ability than HHP treatment of the eggs. Similar has been observed in the zebrafish (*Danio rerio*) eggs where differences in the expression of maternal genes in eggs from different clutches reflected differences between mothers [25].

Genes with transcripts affected on a pointwise level, because of their biological functions, are promising candidates for the further studies on HHP. Although, our statistical analysis cannot fully confirm their involvement in the process of cellular response to HHP, observed minor differences in their expression are suggestive and may give more information and clues for further research than classical typing of the candidate genes. To clarify provided results, expression of the genes whose transcripts differed the most in the control and HHP-treated eggs (Table 2) should be compared using qRT-PCR. These genes are involved in the regulation of development and somatic growth (*1a IGF1RB* and *1b IGF1RB*, *IGF-I* receptor subtype A) [26], response to the DNA damage and regulation of the DNA mismatch repair (*SETD2*, *RIF1*) [27, 28], actin filament polymerization (*FH1/FH2* domain-containing protein 3, *(FHOD3)*), transcriptional regulation (male-specific lethal 3-like 1 *(MS3L1)*), and formation of centromere/kinetochore complex (centromere protein F *(CENPF)*) [29] (Table 2, Supplementary File Data). The *CENPF* gene plays a role in attaching the kinetochore and spindle microtubules [29]. Thus, alterations of the CENPF transcript made by HHP may be a part of the molecular mechanism leading to a damage or dysfunction of the spindle microtubules during cell meiosis and mitosis. It is not excluded that altered transcript levels of *FHOD3* may also be involved in the molecular reaction for HHP leading to the inhibition of cell cleavages in fish. HHP treatment of porcine oocytes resulted in alteration of 44 transcripts [24]. Gene ontology analysis exhibited that altered transcripts played some role during the embryonic development; however, we did not observe any overlap between expression profiles in the HHP-treated porcine and rainbow trout female gametes. Most of the porcine altered transcripts usually showed lower expression levels in the HHP-treated oocytes. Thus, it was suggested that 1 h exposure to HHP promotes precocious degradation of maternal transcriptome [24].

Survivability of salmonid androgenetic and gynogenetic early embryos is lower in eggs that were subjected to the HHP shock [30, 31]. It is not excluded that increased mortality was related to the very discrete changes in the egg cytoplasmic maternal mRNA, though it is more likely that decreased survivability of fish developing in pressurized eggs may be associated with the HHP-induced damages of the cellular organelles and protein structures. Short exposure of fish eggs to HHP depolymerizes microtubules that also play some roles in the transportation of the cytoplasmic particles and factors necessary for the early cellular differentiation of blastomeres [32]. Changes in the microtubule structure caused by HHP may thus disturb fish embryonic development. In crucian carp (*Carassius auratus* Linnaeus 1758), HHP shock administered to inseminated eggs disrupted the proper formation of blastodiscs, impaired the development of cytoplasm, and triggered the delay of epiboly and suppression of the dorsoventral differentiation [9]. Triploid rainbow trout produced using HHP usually exhibits higher incidences of deformities than control diploids [33]; however, it is hard to evaluate which malformations result from the HHP exposure.

## 5. Conclusions

Three-minute exposure to HHP (65.5 MPa) was presumably too short to degrade cytoplasmic RNA and/or significantly alter expression of the maternal transcripts in the rainbow trout eggs. On the other hand, some changes of the maternal transcripts responsible for the embryo growth and development, DNA synthesis and repair, cell divisions, and function of the spindle microtubules, although statistically insignificant, may impair mechanisms crucial for the proper development of the early fish embryos before activation of the zygotic genome.

## Figures and Tables

**Table 1:** RNA integrity and concentration for all RNA purification replicates in the HHP-treated and control eggs.

Replicate	1		2		3		4		5		All	
Homogenization/conservation	Bead mill/RNAlater		Manual/RNAlater		Bead mill/RNAlater		Bead mill/RNAlater		TissueRuptor/dry ice			
Group	Control	HHP	Control	HHP	Control	HHP	Control	HHP	Control	HHP	Control	HHP
Mean RIN	8.90	8.70	7.83	8.03	7.97	8.90	8.80	8.87	8.73	8.93	8.45	8.69
RIN SD	0.14	0.20	0.62	0.45	0.50	0.16	0.14	0.42	0.05	0.12	0.58	0.47
Mean concentration (ng/*μ*l)	224.50	318.43	134.80	122.60	175.87	163.13	178.93	187.17	152.37	164.57	173.29	191.18
Concentration SD (ng/*μ*l)	19.77	72.54	11.21	20.66	19.99	76.60	11.42	96.53	18.89	11.43	34.59	93.18
Min RIN	8.70	8.50	7.30	7.50	7.30	8.70	8.70	8.30	8.70	8.80	7.30	7.50
Max RIN	9.00	8.90	8.70	8.60	8.50	9.10	9.00	9.30	8.80	9.10	9.00	9.30
Min conc.	197.40	220.10	119.60	103.10	148.50	59.30	163.70	55.60	126.90	149.70	119.60	55.60
Max conc.	244.00	392.90	146.30	151.20	195.70	241.80	191.20	284.50	172.10	177.50	244.00	392.90

**Table 2:** The top 10 transcripts with the lowest pointwise *p* value (*p* < 0.01) for the differences in the expression level between HHP-treated and untreated rainbow trout eggs. Downregulated transcripts are written in bold.

Transcript	Gene description	Base mean	Log2 fold change	lfcSE	Stat	*p* value	*p* adj	Treated1	Treated2	Treated3	Untreated1	Untreated2	Untreated3
**C29972_c2_seq1_**	**Predicted: protein transport protein Sec31A-like *(Oreochromis niloticus)***	**807.49**	**−2.19**	**0.76**	**−2.90**	**0.0037**	**1.0**	**1411.93**	**665.51**	**818.05**	**1073.05**	**793.53**	**82.86**
C169673_c0_seq1_	Insulin-like growth factor 1b receptor *(Oncorhynchus kisutch)*	66.73	2.17	0.78	2.80	0.0051	1.0	7.43	48.60	60.68	49.72	35.99	197.95
C63960_c0_seq1_	Predicted: telomere-associated protein RIF1 *(Oreochromis niloticus)*	383.98	2.05	0.73	2.79	0.0053	1.0	95.54	414.58	360.60	230.52	352.88	849.79
C153473_c0_seq1_	IGF-I receptor subtype a *(Cyprinus carpio)*	123.58	2.13	0.77	2.78	0.0054	1.0	22.29	78.35	144.71	91.30	80.76	324.08
C9983_c0_seq1_	Predicted: FH1/FH2 domain-containing protein 3 *(Danio rerio)*	270.05	2.06	0.74	2.78	0.0055	1.0	50.96	325.32	240.40	163.62	313.37	526.63
C93043_c1_seq1_	MAM domain-containing glycosylphosphatidylinositol anchor protein 1 *(Dicentrarchus labrax)*	98.40	2.09	0.76	2.76	0.0058	1.0	11.68	104.14	114.36	89.50	93.05	177.69
C96308_c0_seq1_	Predicted: centromere protein F *(Oreochromis niloticus)*	1329.72	1.85	0.67	2.75	0.0059	1.0	406.59	1473.85	1256.84	1318.94	1195.56	2326.56
C35448_c1_seq1_	Predicted: FH1/FH2 domain-containing protein 3 *(Oreochromis niloticus)*	362.46	1.99	0.75	2.68	0.0075	1.0	77.50	404.66	374.60	158.20	419.59	740.23
C78059_c0_seq1_	Predicted: histone-lysine N-methyltransferase SETD2-like *(Oreochromis niloticus)*	606.50	2.04	0.76	2.67	0.0075	1.0	121.02	847.01	212.39	479.12	565.30	1414.17
**C39441_c0_seq1_**	**Male-specific lethal 3-like 1 *(Salmo salar)* gb**	**217.77**	**−1.92**	**0.72**	**−2.66**	**0.0077**	**1.0**	**350.33**	**259.86**	**179.72**	**280.24**	**191.36**	**45.11**

## Supplementary Materials

Supplementary 1 Table S1: sequencing reads and mapping statistics of eggs originating from three females (F1–F3) treated with HHP and untreated (control, C). Figure S1: exemplary TapeStation trace for RNA provided from the control (A) and HHP-treated (B) eggs. Electropherogram shows the separation profile of the selected mRNA samples showing from the left: lower marker (50 bp, preset in the sample buffer) and 18S and 28S rRNA peaks.

Supplementary 2 File Data: results of expression analysis of rainbow trout eggs subjected to the high hydrostatic pressure (HHP) shock and untreated eggs.
