# Corrigendum to “Transcriptome Analysis of Rainbow Trout (*Oncorhynchus mykiss*) Eggs Subjected to the High Hydrostatic Pressure Treatment”

**DOI:** 10.1155/2018/1057382

**Published:** 2018-12-26

**Authors:** Artur Gurgul, Klaudia Pawlina-Tyszko, Monika Bugno-Poniewierska, Tomasz Szmatoła, Igor Jasielczuk, Stefan Dobosz, Konrad Ocalewicz

**Affiliations:** ^1^Department of Animal Molecular Biology, National Research Institute of Animal Production, Krakowska 1, 32-083 Balice, Poland; ^2^Institute of Veterinary Sciences, University of Agriculture in Krakow, Mickiewicza 24/28 Av, 30-059 Krakow, Poland; ^3^Department of Salmonid Research, Inland Fisheries Institute in Olsztyn, Rutki, 83-330 Żukowo, Poland; ^4^Department of Marine Biology and Ecology, Institute of Oceanography, Faculty of Oceanography and Geography, University of Gdansk, M. Piłsudskiego 46 Av, 81-378 Gdynia, Poland

In the article titled “Transcriptome Analysis of Rainbow Trout (*Oncorhynchus mykiss*) Eggs Subjected to the High Hydrostatic Pressure Treatment” [1], there was an error in the first affiliation, where “Laboratory of Genomics, Department of Animal Genetics and Molecular Biology” should be changed to “Department of Animal Molecular Biology.” The corrected affiliation is shown above.
