# Supplementary material for: Transcriptome Analysis of Rainbow Trout (Oncorhynchus mykiss) Eggs Subjected to the High Hydrostatic Pressure Treatment
Source: Int J Genomics. 2018 Aug 26;2018:5197126. doi: 10.1155/2018/5197126 (PMC6129359; doi:10.1155/2018/5197126)
Supplement: Supplementary 1 — Table S1: sequencing reads and mapping statistics of eggs originating from three females (F1–F3) treated with HHP and untreated (control, C). Figure S1: exemplary TapeStation trace for RNA provided from the control (A) and HHP-treated (B) eggs. Electropherogram shows the separation profile of the selected mRNA samples showing from the left: lower marker (50 bp, preset in the sample buffer) and 18S and 28S rRNA peaks. [file 5197126.f1.doc]

Supplementary materials

**Supplementary Figure S1.** Exemplary TapeStation trace for RNA provided from the control (A) and HHP treated (B) eggs. Electropherogram shows the separation profile of the selcted mRNA samples showing from the left: lower marker (50bp, preset in the sample buffer), 18S and 28S rRNA peaks.


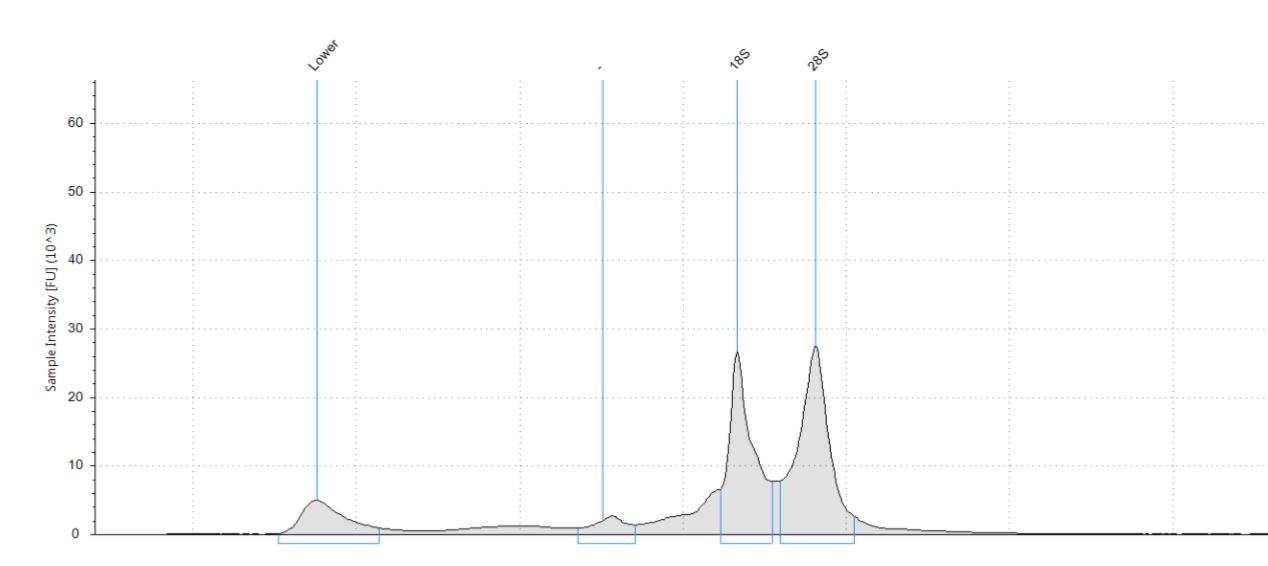
**
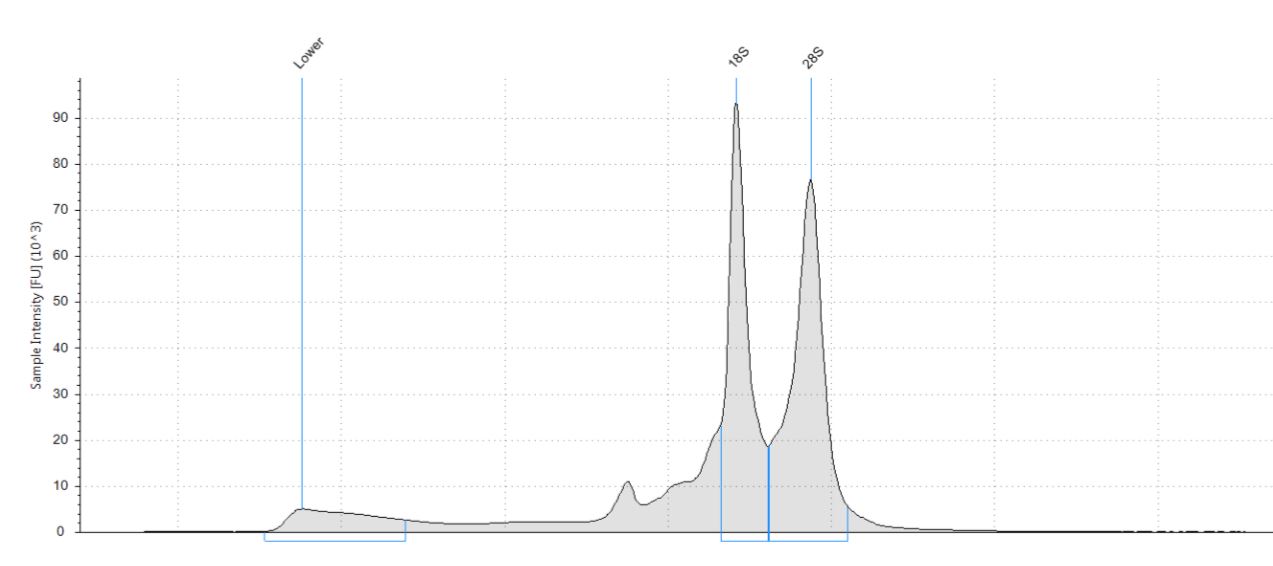
**

**A**

**B**

**Supplementary Table S1.** Sequencing reads and mapping statistics of eggs originating from three females (F_1_ - F_3_) treated with HHP and

untreated (control; C)

| Sample | Number of good reads | Number of reads per group | Number of mapped reads | Number of mapped reads per group | Percentage of mapped reads | Average percentage of mapped reads |
| --- | --- | --- | --- | --- | --- | --- |
| F_1_C | 28,799,246 | 88,359,078 | 17,040,347 | 52,840,849 | 59.17 | 59.80 |
| F_2_C | 29,350,016 |  | 17,761,520 |  | 60.52 |  |
| F_3_C | 30,209,816 |  | 18,038,982 |  | 59.71 |  |
| F_1_HHP | 24,065,752 | 74,808,457 | 13,725,410 | 44,595,699 | 57.03 | 59.57 |
| F_2_HHP | 25,340,078 |  | 15,338,765 |  | 60.53 |  |
| F_3_HHP | 25,402,627 |  | 15,531,524 |  | 61.14 |  |
| All samples | 163,167,535 |  | 97,436,548 |  | 59.68 |  |
